# Is Afamin a novel biomarker for gestational diabetes mellitus? A pilot study

**DOI:** 10.1186/s12958-018-0338-x

**Published:** 2018-03-27

**Authors:** Angela Köninger, Annette Mathan, Pawel Mach, Mirjam Frank, Boerge Schmidt, Ekkehard Schleussner, Rainer Kimmig, Alexandra Gellhaus, Hans Dieplinger

**Affiliations:** 1 Department of Gynecology and Obstetrics, University of Duisburg-Essen, Hufelandstrasse 55, 45122 Essen, Germany; 2 Department of Gynecology and Obstetrics, Klinikum Würzburg Mitte, Salvatorstrasse 7, 97074 Würzburg, Germany; 3 Institute for Medical Informatics, Biometry and Epidemiology (IMIBE), University of Duisburg-Essen, Hufelandstrasse 55, 45122 Essen, Germany; 4 Department of Obstetrics, Jena University Hospital, Am Klinikum 1, 07747 Jena, Germany; 5 Division of Genetic Epidemiology, Department of Medical Genetics, Molecular and Clinical Pharmacology, Medical University of Innsbruck, Schöpfstrasse 41, 6020 Innsbruck, Austria; 6 Vitateq Biotechnology GmbH, 6020 Innsbruck, Austria

**Keywords:** Afamin, Pregnancy, Insulin resistance, Gestational diabetes mellitus, Oral glucose tolerance test

## Abstract

**Background:**

In search of potential early biomarkers for timely prediction of gestational diabetes mellitus (GDM), we focused on afamin, a vitamin E–binding protein in human plasma.. Afamin plays a role in anti-apoptotic cellular processes related to oxidative stress and is associated with insulin resistance and other features of metabolic syndrome. During uncomplicated pregnancy its serum concentrations increase linearly. The aim of this study was to investigate the suitability of afamin as early marker for predicting GDM.

**Methods:**

In a first-trimester cohort from a prospective observational study of adverse pregnancy outcomes we secondarily analyzed afamin concentrations in 59 patients diagnosed with GDM and 51 controls. Additionally, afamin concentrations were cross-sectionally examined in a mid-trimester cohort of 105 women and compared with results from a simultaneously performed oral glucose tolerance test (OGTT). Subgroup analysis comparing patients treated with either insulin (iGDM) or dietary intervention (dGDM) was performed in both cohorts. Patients were recruited at the University Hospital Essen, Germany, between 2003 and 2016.

**Results:**

Results were adjusted for body-mass-index (BMI) and gestational age. First and mid-trimester cohorts yielded significantly elevated afamin concentrations in patients with pathological OGTT compared to patients without GDM (first trimester cohort: mean, 113.4 mg/l; 95% CI, 106.4–120.5 mg/l and 87.2 mg/l; 95% CI, 79.7–94.7 mg/l; mid-trimester cohort: mean, 182.9 mg/l; 95% CI, 169.6–196.2 mg/l and 157.3 mg/l; 95% CI, 149.1–165.4 mg/l, respectively). In the first-trimester cohort, patients developing iGDM later in pregnancy presented with significantly higher afamin concentrations compared to patients developing dGDM and compared to patients without GDM. In the mid-trimester cohort, mean concentrations of afamin differed significantly between patients with dGDM compared to controls and between patients with iGDM and controls. Patients with iGDM showed only slightly higher afamin levels compared to patients with dGDM.

**Conclusion:**

Afamin may serve as a new early biomarker for pathological glucose metabolism during pregnancy. Further research is needed to determine afamin’s concentrations during pregnancy, its predictive value for early detection of pregnancies at high risk to develop GDM and its diagnostic role during the second trimester.

## Background

Gestational diabetes mellitus (GDM) is a common disorder that occurs in approximately 7% to 14% of pregnancies [1]. GDM is defined as “any degree of glucose intolerance with onset or first recognition during pregnancy” [2].

The prevalence of GDM depends strongly on ethnicity and the diagnostic criteria used [3]. The absence of a worldwide standard concerning the best criteria for diagnosing GDM results in unequal management concepts, variances in incidence rates, and a lack of scientific evidence of the consequences for the mother and the baby. As shown by the Hyperglycemia and Adverse Pregnancy Outcome (HAPO) trial, pathological findings from the 75-g oral glucose tolerance test (OGTT) are strongly associated with adverse pregnancy outcome [4, 5]. Some studies have demonstrated a benefit for the mother and the baby if diabetes is screened, diagnosed, and treated during pregnancy [6, 7]. As shown by Crowther et al. [7], the number of patients who need to be treated to prevent one serious event, such as shoulder dystocia, a high-grade birth injury such as nerve palsy, bone fracture, or neonatal death, is approximately 34. An important finding in Landon’s [6] interventional trial involving patients with mild GDM was a reduction in the risk of cesarean section and of preeclampsia if GDM was screened, diagnosed, and treated. In summary, the exact and timely diagnosis of GDM allows successful interventions and improves pregnancy outcomes.

The 75-g OGTT is currently considered to be the gold standard for diagnosing GDM [8]. However, the test presents some problems in performance and pre-analytic requirements, such as the need for patients to be in a fasting state, the need to avoid glycolysis of the samples, and the need to perform laboratory analysis in accordance with highly standardized analysis procedures and low inter-assay variations. Some countries recommend screening tests for determining which pregnant women may be at high risk of GDM; these tests include the 50-g OGTT, which was performed in the two interventional studies reported thus far [6, 7, 9]. However, this test has limitations in terms of sensitivity, specificity, and reproducibility [10, 11]. For these reasons, the consensus statement of the *International Association of the Diabetes and Pregnancy Study Groups* (IADPSG) [8] recommends that “in future clinical practice, simpler and more cost-effective strategies that do not require performing an OGTT on most pregnant women may be developed”.

In the current study, our search for potential new and early biomarkers that can offer timely prediction of GDM focused on afamin, a previously described vitamin E–binding protein found in human plasma [12]. Vitamin E is an important antioxidant that protects against oxidative stress. Afamin is a member of the albumin gene family [13] that seems to play a role in anti-apoptotic cellular processes related to oxidative stress [14]. Plasma concentrations of afamin are independent of fasting status, age, and sex [15, 16] and increase linearly approximately 2-fold during an uncomplicated pregnancy [17]. Afamin concentrations are strongly correlated with clinical and laboratory parameters of the metabolic syndrome, such as elevations in body mass index (BMI) and plasma glucose concentrations, dyslipidemia, and hypertension [18]. It was subsequently shown in a very recent population-based study in more than 20,000 individuals that afamin concentrations are also associated with the prevalence and incidence of type 2 diabetes mellitus as well as insulin resistance (IR) [19].

As we have previously shown, afamin concentrations are also associated with the presence of IR among patients with polycystic ovary syndrome (PCOS) [20]. IR can be determined by the Homeostasis-Model Assessment (HOMA) [21]. Physiologically, IR increases during pregnancy with the aim of adequate maternofetal glucose transfer. Increased IR at the beginning of pregnancy is strongly associated with the development of GDM [22]. Additionally, insulin concentrations regulate the expression of sex hormone–binding protein (SHBG) in the liver [23] and low concentrations of SHBG during the first trimester of pregnancy may indicate the development of GDM [24]. However, no reliable method of screening for diabetes during the first trimester currently exists.

The aim of this study was to explore the predictive and diagnostic value of afamin concentrations regarding GDM, in the first and second trimester, respectively. In a secondary analysis of samples which were taken from a prospective observational study with the aim to predict adverse pregnancy outcomes, we examined the association between serum afamin concentrations during the first trimester and the subsequent development of GDM during the ongoing pregnancy. In a mid-trimester cross-sectional analysis we also examined serum afamin concentrations in serum samples from women with and without a pathological 75-g OGTT in median gestational age of 26th week of pregnancy.

## Methods

### Subjects characteristics

#### First-trimester cohort

Frozen serum samples from 110 women with singleton pregnancies in the first trimester of pregnancy were secondarily analyzed. We included all patients who gave birth at the Department of Obstetrics and Gynecology of the University Hospital of Essen between 2003 and 2014 with either a GDM diagnosis at the time of delivery or an uncomplicated pregnancy and from whom frozen blood samples collected during the first trimester were available. 59/110 developed GDM in the second trimester and 51/110 did not develop GDM and served as controls. Patients were 19 to 44 years old (median, 34 years; interquartile range [IQR], 31–37 years) and presented between gestational day 43 and 98 (median 89 days of pregnancy [IQR 85–92]). 8 patients from the cohort, all with GDM, had confirmed polycystic ovarian syndrome (PCOS) corresponding to the prevalence of PCOS in the general population [25].

Information about GDM was available from our medical records or from patient interviews. GDM was diagnosed by the women’s gynecologists according to the current guidelines for obstetric care in Germany [9]. GDM is diagnosed if at least one value of the 75 g-OGTT was elevated. Before 2011, the following definition was used: fasting glucose: 95 mg/dl, glucose after 1 h: 180 mg/dl, glucose after 2 h: 155 mg/dl [9, 26]. The values are based on maternal life-time risk for diabetes [26]. Since 2011, the following definition is used in Germany to diagnose GDM: fasting glucose: 92 mg/dl, glucose after 1 h: 180 mg/dl, glucose after 2 h: 153 mg/dl [4, 9, 26]. These values are based on adverse pregnancy outcomes (HAPO trial 2008). 36 patients with GDM presented before 2011 and 23 after 2011.

According to current guidelines, only patients at elevated risk for GDM e.g. obesity, family history with diabetes or macrosomia in a pregnancy before or patients with a pathological 50 g-OGTT got a 75 g-OGTT before 2013. Since 2013 to the end of the study period, the performance of a 50 g-OGTT as a screening tool for GDM was a standard procedure in Germany. Patients with either glucose levels ≥135 mg/dl in the 50 g-OGTT or patients with risk factors got a 75 g-OGTT [9]. Consequently, not all pregnant women in Germany got a 75 g-OGTT. Since we performed a secondary analysis using data from clinical routine, a 75 g-OGTT was not available from all patients of the control group because they were screened according to current guidelines and after screening OGTT was not indicated for all of the controls.

#### Mid-trimester cohort

We also performed a cross-sectional analysis involving 105 pregnant women with singleton pregnancies aged 20 to 43 years (median, 32 years; IQR, 28–36 years). Between 2014 and 2016, women were enrolled in the study as they presented to the University Hospital of Essen Department of Obstetrics and Gynecology for antenatal care in high-risk pregnancies. 5/107 suffered from PCOS. Patients underwent a 75-g OGTT for GDM screening between gestational day 44 and 272 (median 184; IQR 162–209). Simultaneously with the test, blood samples were collected for afamin determination. Patients in whom the test was performed before 24 weeks and with no pathological results in the OGTT, received another OGTT after reaching the 24th week of pregnancy. If both results differed from each other, they were excluded from the study. OGTT was performed according to the practice guideline of the German Diabetes Association (DDG) and the German Association for Gynecology and Obstetrics (DGGG) [9]. To avoid glycolysis, we transported venous blood samples to the laboratory of the University Hospital Essen within 15 min after sampling. Fasting insulin concentrations were determined at presentation (0 h), after 1 h, and after 2 h. Insulin resistance was defined according to HOMA [20]. Serum collected while patients were fasting was also used to determine afamin concentrations. All women of both cohorts provided written informed consent, and the study was approved by the local research ethics committee (number 125212-BO).

### Blood sampling

First trimester cohort: 9 ml of blood was drawn with the S-Monovette Blood Collection System (Sarstedt AG and Co., Nürnbrecht, Germany) for freezing and later determination of parameters of interest. Samples were stored immediately at 4 °C and processed within 4 h to avoid cell lysis. Blood fractionation was carried out by centrifugation at 2500×g for 10 min, and 3 to 4 ml of the supernatant constituting blood serum was removed and stored at − 80 °C. For afamin analysis, all samples were thawed to divide them into aliquots and restored at − 80 °C. Frozen aliquots were sent to the Medical University of Innsbruck, Division of Genetic Epidemiology in 2016 for determination of afamin concentrations.

Mid-trimester cohort: 2.7 ml blood were collected into 2.7-ml fluoride/EDTA monovettes for analyzing glucose concentrations. The S-Monovette Blood Collection System (Sarstedt AG and Co., Nürnbrecht, Germany) was used to collect 9 ml blood for insulin determination. Glucose and insulin concentrations were immediately determined in the central laboratories of the University Hospital Essen. 9 ml of blood was also drawn with the S-Monovette Blood Collection System (Sarstedt AG and Co., Nürnbrecht, Germany) for determination of afamin. These samples were stored at 4 °C immediately and processed within 4 h to avoid cell lysis. Blood fractionation was carried out by centrifugation at 2500×g for 10 min, and 3 to 4 ml of the supernatant constituting blood serum was removed and stored at − 80 °C. For afamin analysis all samples were thawed to divide them into aliquots and restored at − 80 °C. Frozen samples were sent to the Medical University of Innsbruck, Division of Genetic Epidemiology in 2016 for determination of afamin concentrations.

### Laboratory parameters

Afamin concentrations were measured with a commercially available sandwich enzyme-linked immunosorbent assay (ELISA; BioVendor, Brno, Czech Republic) using two different monoclonal antibodies against human afamin as modified from a previously described protocol [15]. Recombinantly expressed and purified human afamin served as the assay standard. According to the manufacturer’s manual, within-run and run-to-run coefficients of variation were 3.6% and 3.4%, respectively, at a mean afamin concentration of 80 mg/l.

Glucose concentrations were determined photometrically (ADVIA Centaur CP Immunoassay System; Siemens Healthcare Diagnostics, Eschborn, Germany). Analyses were performed according to the guidelines of the German Medical Association (www.bundesaerztekammer.de). Automated chemiluminescence immunoassay systems (Immulite 2000 XPi; Siemens Healthcare Diagnostics) were used to determine insulin concentrations. Intra-assay variations were ≤1.1% for glucose and ≤5.5% for insulin; inter-assay variations were ≤1.8% for glucose and ≤7.3% for insulin.

### Statistical analysis

Characteristics of both study populations were presented as mean with standard deviations (SD) as well as medians with interquartile ranges (IQR). Mann-Whitney-U-Test or t-test were used to analyse group differences in maternal age, gestational age, BMI, newborn weight, gestational age at birth, afamin, glucose and insulin concentrations and HOMA-IR. In both cohorts, differences in afamin concentrations between subgroups were analyzed using linear regression models including gestational age and BMI at the time of blood sampling as covariate and status of iGDM or dGDM as categorical independent variables to estimate gestational age- and BMI -adjusted least-squares means with 95% confidence intervals (CIs) as marginal averages. The alpha level was set at 0.05 to determine statistical significance. All analyses were performed with the R statistical package, version 3.0.2 [27].

## Results

### First-trimester study population

Patient characteristics (*n* = 110) are shown in Table 1. Of the 59 participants who had GDM diagnosis later in their pregnancy, 25 were treated with dietary intervention (dGDM) and 34 were treated with insulin (iGDM). Patient characteristics of the subgroups are shown in Table 2.

**Table 1:** Patients’ characteristics and afamin concentrations in the first-trimester cohort study

Parameter	Controls n = 51	Gestational diabetes mellitus (GDM) *n* = 59	*p*-value
Maternal age (years)			
Mean (± standard deviation, SD)	32.65 (4.79)	34.36 (5.26)	0.05
Median (Interquartile range, IQR)	33.00 (29.25–36.00)	36.00 (32.00–38.00)	
Body-mass-index (kg/m^2^)			
Mean (± SD)	24.76 (5.00)	30.05 (8.02)	< 0.001
Median (IQR)	24.00 (21.80–26.85)	28.85 (24.40–34.30)	
Afamin (mg/l)			
Mean (± SD)	84.59 (17.75)	115.46 (31.72)	< 0.001
Median (IQR)	82.18 (68.68–100.57)	117.13 (86.27–146.99)	
Newborn weight (gram)			
Mean (± SD)	3517 (453)	3148 (730)	0.03
Median (IQR)	3490 (3270–3865)	3320 (2868–3568)	
Gestational age at parturition (days)			
Mean (± SD)	276 (8)	266 (21)	0.02
Median (IQR)	278 (272–282)	273 (262–280)

**Table 2:** Patients’ characteristics and afamin concentrations in the first-trimester cohort study of patients with diagnosis of gestational diabetes mellitus (GDM) later in pregnancy

Parameter	Gestational diabetes mellitus treated with dietary intervention (dGDM) n = 25	Gestational diabetes mellitus treated with insulin (iGDM) n = 34	*p*-value
Maternal age (years)			
Mean (± standard deviation, SD)	33.60 (6.37)	34.91 (4.29)	0.50
Median (Interquartile range, IQR)	36.00 (28.50–37.25)	36.00 (32.00–39.00)	
Body-mass-index (kg/m^2^)			
Mean (± SD)	26.19 (5.98)	32.78 (8.23)	0.002
Median (IQR)	24.85 (22.10–30.80)	29.50 (27.90–37.40)	
Afamin (mg/l)			
Mean (± SD)	91.33 (21.43)	133.20 (25.87)	< 0.001
Median (IQR)	86.99 (75.88–100.94)	140.18 (118.20–154.70)	
Newborn weight (gram)			
Mean (± SD)	3376 (709)	2985 (711)	0.008
Median (IQR)	3510 (3165–3680)	3105 (2768–3453)	
Gestational age at parturition (days)			
Mean (± SD)	269 (20)	264 (21)	0.25
Median (IQR)	277 (266–281)	272 (251–280)	

Since afamin levels depend on BMI and gestational age, the subsequent results comparing afamin levels between controls, iGDM and dGDM patients, were adjusted for BMI and gestational age:

Serum afamin concentrations measured during the first trimester were significantly higher among patients with GDM (mean, 113.4 mg/l; 95% CI, 106.4–120.5 mg/l) than among patients without GDM diagnosis later in their pregnancy (mean, 87.2 mg/l; 95% CI, 79.7–94.7 mg/l; *P* = < 0.0001).

**Fig. 1:**
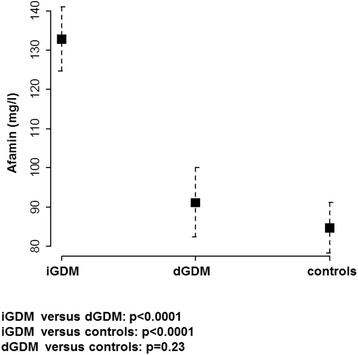
Afamin concentrations in the first trimester cohort (between gestational day 8 and 98). Serum concentrations of afamin among patients with gestational diabetes mellitus (GDM) treated with insulin (iGDM; *n* = 34), treated with dietary intervention (dGDM; *n* = 25) and control subjects (*n* = 51) adjusted for gestational age and body-mass-index (BMI)

Patients which developed iGDM (mean, 132.8 mg/l; 95% CI, 124.6–141.0 mg/l) presented with significantly higher afamin concentrations than patients which developed dGDM (mean, 91.2 mg/l; 95% CI, 82.4–100.0 mg/l; *P* < 0.0001) and than patients without GDM in their ongoing pregnancy (mean, 84.7 mg/l; 95% CI, 78.4–91.1 mg/l; *P* < 0.0001). No significant difference was seen between afamin concentrations of patients with dGDM in their later pregnancy and controls (*p* = 0.23) (Fig. 1).

### Mid-trimester cohort

Patient characteristics are shown in Table 3. Of the 105 patients, 29 exhibited abnormal OGTT results and 76 exhibited normal OGTT results. Of the 29 patients with GDM, 12 were treated with insulin (iGDM) and 17 with dietary intervention (dGDM). Patient characteristics of the two subgroups are shown in Table 4.

**Table 3:** Patients´ characteristics and laboratory parameters in the mid-trimester cohort study

Parameter	Normal 75 g-oral glucose-tolerance-test (OGTT) n = 76	Pathological 75 g-oral glucose-tolerance-test (OGTT) *n* = 29	*p*-value
Gestational age at 75 g-oral glucose-tolerance-test (OGTT) performance (days)			
Mean (± standard deviation, SD)	189 (40)	168 (43)	0.06
Median (Interquartile range, IQR)	189 (165–221)	176 (151.25–200.00)	
Maternal age (years)			
Mean (± SD)	30.59 (5.08)	35.00 (5.01)	< 0.001
Median (IQR)	30.50 (27.50–34.00)	37.00 (31.50–39.00)	
Body-mass-index (kg/m^2^)			
Mean (± SD)	28.35 (7.13)	31.15 (8.56)	0.08
Median (IQR)	26.80 (23.40–32.00)	29.30 (25.60–34.65)	
Fasting Insulin (μlU/ml)			
Mean (± SD)	9.68 (7.53)	12.68 (7.94)	0.02
Median (IQR)	7.90 (4.40–12.25)	10.00 (7.63–14.90)	
1 h Insulin (μlU/ml)			
Mean (± SD)	90.26 (54.17)	110.45 (57.89)	0.07
Median (IQR)	76.30 (52.35–121.00)	102.00 (68.23–123.50)	
2 h Insulin (μlU/ml)			
Mean (± SD)	69.76 (46.40)	121.16 (54.21)	< 0.001
Median (IQR)	58.15 (42.90–85.10)	120.00 (84.63–148.25)	
Fasting glucose (mg/dl)			
Mean (± SD)	77.70 (5.68)	83.79 (11.93)	0.03
Median (IQR)	77.00 (74.00–81.50)	81.00 (73.75–93.25)	
1 h glucose (mg/dl)			
Mean (± SD)	135.25 (24.86)	179.00 (25.97)	< 0.001
Median (IQR)	136.50 (119–156.50)	175.00 (161.00–189.25)	
2 h glucose (mg/dl)			
Mean (± SD)	105.04 (21.02)	152.69 (26.02)	< 0.001
Median (IQR)	101.50 (87.00–121.00)	156.00 (148.25–170.25)	
HOMA-IR			
Mean (± SD)	1.90 (1.52)	2.72 (1.96)	0.01
Median (IQR)	1.50 (0.80–2.45)	2.20 (1.53–3.33)	
Afamin (mg/l)			
Mean (± SD)	157.90 (36.32)	180.83 (41.21)	0.003
Median (IQR)	160.49 (142.63–177.33)	180.20 (158.68–211.94)	
Gestational age at parturition (days)			
Mean (± SD)	259 (21)	256 (22)	0.38
Median (IQR)	266 (249–273)	263 (249–269)	
Newborn weight (gram)			
Mean (± SD)	2851 (786)	2916 (821)	0.71
Median (IQR)	3075 (2465–3385)	3170 (2496–3473)

**Table 4:** Gestational diabetes mellitus (GDM) patients´ characteristics and laboratory parameters in the mid-trimester cohort study

Parameter	Gestational diabetes mellitus treated with dietary intervention (dGDM) n = 17	Gestational diabetes mellitus treated with insulin (iGDM) n = 12	*p*-value
Gestational age at 75 g-oral glucose-tolerance-test (OGTT) performance (days)			
Mean (± standard deviation, SD)	174 (32)	158 (55)	0.32
Median (Interquartile range, IQR)	183 (157–200)	167 (120–199)	
Maternal age (years)			
Mean (± SD)	34.59 (4.64)	35.58 (5.85)	0.40
Median (IQR)	36 (32.25–38.00)	37.50 (30.00–39.50)	
Body-mass-index (kg/m^2^)			
Mean (± SD)	28.05 (5.28)	35.53 (10.49)	0.02
Median (IQR)	26.60 (24.55–31.33)	30.50 (29.70–38.90)	
Fasting Insulin (μlU/ml)			
Mean (± SD)	9.95 (5.04)	16.53 (9.79)	0.06
Median (IQR)	9.90 (5.48–14.30)	13.40 (9.35–20.35)	
1 h Insulin (μlU/ml)			
Mean (± SD)	82.04 (24.47)	150.69 (68.15)	0.003
Median (IQR)	75.50 (63.98–103.25)	125.00 (111.50–213.00)	
2 h Insulin (μlU/ml)			
Mean (± SD)	101.59 (40.58)	148.88 (60.45)	0.02
Median (IQR)	102.00 (71.80–123.50)	141.00 (107.50–187.50)	
Fasting glucose (mg/dl)			
Mean (± SD)	79.47 (9.27)	89.92 (12.93)	0.02
Median (IQR)	77.00 (73.00–89.25)	93.50 (82.00–98.00)	
1 h glucose (mg/dl)			
Mean (± SD)	171.53 (16.90)	189.58 (33.07)	0.14
Median (IQR)	171.00 (160.25–187.50)	183.50 (171.50–202.50)	
2 h glucose (mg/dl)			
Mean (± SD)	155.35 (20.34)	148.92 (33.08)	0.81
Median (IQR)	155.00 (151.25–172.00)	157.00 (135.00–169.00)	
HOMA-IR			
Mean (± SD)	2.00 (1.07)	3.75 (2.47)	0.04
Median (IQR)	1.80 (0.98–2.98)	2.90 (1.90–4.90)	
Afamin (mg/l)			
Mean (± SD)	176.40 (48.33)	187.10 (29.19)	0.50
Median (IQR)	175.80 (158.68–206.39)	184.72 (160.23–214.95)	
Gestational age at parturition (days)			
Mean (± SD)	257 (26)	256 (14)	0.33
Median (IQR)	266 (252–270)	260 (243–267)	
Newborn weight (gram)			
Mean (± SD)	2935 (945)	2889 (644)	0.89
Median (IQR)	3170 (2570–3505)	2915 (2303–3448)	

When the analysis was adjusted for gestational age and BMI, the differences in afamin concentrations were significant between GDM patients (mean, 182.9 mg/l; 95% CI, 169.6–196.2 mg/l) and control subjects (mean, 157.3 mg/l; 95% CI, 149.1–165.4 mg/l; *P* = 0.002).

**Fig. 2:**
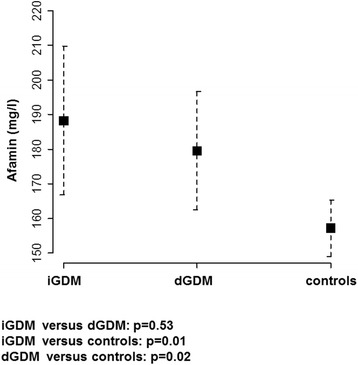
Afamin concentrations in the mid-trimester cohort (between gestational day 44 and 272). Serum concentrations of afamin among patients with gestational diabetes mellitus (GDM) treated with insulin (iGDM; *n* = 12), treated with dietary intervention (dGDM; *n* = 17) and control subjects (*n* = 76) adjusted for gestational age and body-mass-index (BMI)

Mean concentrations of afamin differed significantly between control subjects (mean, 157.2 mg/l; 95% CI, 149.0–165.4 mg/l) and dGDM patients (mean, 179.5 mg/l; 95% CI, 162.5–196.5 mg/l; *P* = 0.02) as well as patients with iGDM (mean, 188.2 mg/l; 95% CI, 166.8–209.7 mg/l; *P* = 0.01). Patients with iGDM showed only slightly higher afamin levels compared to patients with dGDM (*P* = 0.53) (Fig. 2).

HOMA-IR was significantly higher in patients with iGDM (median 2.90, IQR 1.78–5.75) compared to controls (median 1.50; IQR 0.77–2.48; *P* = 0.001) and compared to patients with dGDM (median 1.80; IQR 0.95–3.05; *P* = 0.04). The difference between HOMA-IR of patients with dGDM and controls was not significant (*P* = 0.34).

## Discussion

The aim of this pilot study was to evaluate new screening or diagnostic tools for GDM. Currently, no screening tool exists that can be used during the first trimester of pregnancy to determine which patients will experience GDM during the course of pregnancy. The gold standard for diagnosing GDM during the second trimester is the 75-g OGTT [4, 5]. The corresponding trial showed that an increase in each of the measured values (fasting glucose concentrations at baseline and glucose concentrations after 1 and 2 h) was positively correlated with the risk of adverse outcome. These findings highlight the importance of a carefully performed 75-g OGTT. However, the 75-g OGTT requires high quality in pre-analytic steps, analysis, performance and requires patients to be in a fasting state. For these reasons, the 75-g OGTT cannot be offered to all pregnant women. In addition, the 50-g OGTT, an established screening method for determining which women are at risk of GDM, lacks sensitivity and specificity [10, 11]. Therefore, new and effective screening and diagnostic tools for GDM are recommended [8].

In the current study, we analysed serum concentrations of afamin in samples collected from 215 pregnant women. Because afamin concentrations are known to increase during the course of pregnancy [17] and depend on BMI [18], we adjusted the analysis for gestational age and BMI. Women which developed GDM presented with significantly higher afamin concentrations during the first trimester than patients without GDM in their ongoing pregnancy.

Women with subsequent iGDM presented higher serum afamin concentrations during the first trimester than women with subsequent dGDM or women free of GDM. Women which developed dGDM showed only slightly higher afamin levels in the first trimester compared to women free of GDM. Since in the first trimester cohort the blood samples were secondarily collected in a prospective observational study between 2003 and 2014, not all of the participants received a 75 g-OGTT because in Germany, 75 g-OGTT was only recommended in pregnant women at elevated risk for GDM or with a pathological 50 g-OGTT until 2017. Therefore, patients with undiagnosed GDM may be part of the control cohort. This may explain the comparable results in the control and the dGDM group. In the cohort of GDM patients who had confirmed GDM, however, a significant difference in afamin concentrations was found between patients with iGDM and patients who did not need insulin therapy.

In a mid-trimester cohort we evaluated cross-sectionally the ability of serum afamin concentrations to predict pathological results from a 75-g OGTT. We analyzed serum afamin concentrations among 105 patients undergoing 75-g OGTT with a median gestational age of 26th weeks of pregnancy. We found that serum afamin concentrations were significantly higher among patients with pathological findings by OGTT than among control subjects.

Additionally, serum afamin concentrations, determined when the 75 g-OGTT was performed, were higher among iGDM than among dGDM patients without reaching statistical significance. This is in contrast to the results from the first trimester group, but may be due to the smaller subgroups. We also determined HOMA-IR in the subgroups since afamin levels are known to correlate with HOMA-IR [18, 20]. The HOMA-IR was also significantly higher among patients with pathological OGTT. HOMA-IR differed significantly between iGDM patients and controls and between iGDM patients and dGDM patients, but no difference was observed between patients with dGDM compared to controls. This is in contrast to the result of a significant difference in afamin values between dGDM patients and controls. However, the study design was not suitable to compare the diagnostic accuracy of HOMA-IR and afamin values to predict insulin dependence. Further research with a larger sample size is recommended to explore the role of afamin concentrations, the HOMA-IR or the combination of both parameters in the prediction of iGDM or dGDM in the second trimester.

Because afamin concentrations increase during the course of uncomplicated pregnancies [17] and because OGTT is a test routinely performed between 24 and 28 weeks of gestation, our study design could not determine a cut-off value indicating pathological conditions that exactly corresponded with gestational age.

In all investigated subgroups, including individuals with uncomplicated pregnancies, we found surprisingly high concentrations of afamin, considerably higher than those measured in the general population and pregnant women from other ethnic origins [15, 17]. The ethnic background seems to play a (not yet widely investigated) role in determining afamin concentrations since a previous study from individuals from the same regional origin had also markedly higher average afamin concentrations compared to previously published work analyzing afamin concentrations from different regions [20].

Afamin is known as an indicator of oxidative stress [18]. Oxidative stress itself is strongly associated with IR and with obesity [28, 29]. Previous studies have shown that higher serum concentrations of afamin are associated with IR and with elevated glucose concentrations [18–20]. During pregnancy, the severity of IR increases with ongoing gestational age [30]. The physiological role of the decrease in insulin sensitivity appears to be the appropriate materno-fetal transfer of glucose. Interestingly, IR increases similarly in every woman independent of preconception IR or obesity [22]. As a consequence, women with elevated IR concentrations before conception experience pathologic glucose metabolism during pregnancy, and this condition results in pathologic materno-fetal glucose transfer followed by adverse pregnancy outcomes. Mediators of the increasing severity of IR are produced by the placenta [31]. As shown by Kirwan et al. [32], tumor necrosis factor alpha (TNF-α) plays the most important role in physiologic IR. Adipose tissue produces a cytokine pattern nearly identical to that of the placenta [33]. This condition explains the fact that obese women are at higher risk of GDM. However, concentrations of mediators of IR, such as TNF-α, are also known to be increased during conditions of oxidative stress [34]. TNF-α itself inhibits insulin activity [35]. A well-known condition of IR associated with the presence of mediators of oxidative stress is PCOS [34, 36]. With regard to the association between oxidative stress and glucose metabolism, afamin concentrations seem to indicate the presence of IR among PCOS patients, as we have previously shown [20].

The present study showed that afamin concentrations are significantly higher among pregnant patients with pathological findings of 75-g OGTT. Increased afamin concentrations during the first trimester also indicated the development of iGDM. Afamin seems to be able to early predict a pathologic glucose metabolism during pregnancy. In accordance with the pathomechanisms of GDM, serum afamin concentrations probably reflect situations in which increased IR and oxidative stress result in GDM. The finding that afamin concentrations are higher among iGDM patients than among dGDM patients indicates that high afamin concentrations are also associated with increased severity of IR.

Because tests for serum afamin concentrations do not require the patient to be in a fasting state, these concentrations are very suitable as a biomarker of GDM [15, 16]. Until 2017, the 50-g OGTT was established as a screening method for GDM [6, 9]. In addition to limitations in the accuracy of results from this test, the test also requires a high level of personal and standardized procedures. This may be one reason why this method was not routinely integrated into maternal care in Germany before 2013 [8].

In accordance with the recommendations of the IADPSG in searching for “simpler and more cost-effective strategies that do not require performing an OGTT,” serum afamin concentrations may be a very suitable and easily applicable biomarker that can be used to diagnose patients for GDM during the second trimester of pregnancy or even to screen patients in high risk to develop GDM as early as possible during the first trimester.

However, the use of serum afamin concentrations has also limitations. Because high afamin concentrations indicate IR, the results may be non-specific in detecting GDM. Other conditions, such as preeclampsia [17] or preterm labor, are also associated with IR [37, 38]. Furthermore, the first trimester part of the current study may also have limitations: screening for GDM with a 50 g- or 75 g-OGTT was not a standard procedure in Germany before 2013. Therefore, the control group in our first-trimester cohort consisted of patients without GDM diagnosis at birth according to currently available guidelines, but undiagnosed GDM cannot be completely excluded. In 2005, the prevalence of GDM was 2.5% and in 2015, it was 13.2% [39]. This increased prevalence is most likely a consequence of the changed methods of GDM screening and reflects the situation in Germany. For these reasons, we did not determine a first-trimester afamin cut-off value that distinguishes patients developing GDM or not in their ongoing pregnancy in this pilot study. Additionally, we performed no subgroup analysis to the slightly differing thresholds of the 75 g-OGTT before and after 2011.

The cross-sectional part of our study did not require administration of the OGTT at the identical gestational age for every patient. Our analysis of afamin concentrations was adjusted for gestational age because afamin concentrations increase during the course of pregnancy, but our study design did not allow us to determine cut-off values for each gestational week. The mean gestational age at parturition was 36 weeks indicating the high-risk group. However, this is a pilot study presenting first data of afamin concentrations in correlation with pathological 75 g OGTT.

## Conclusion

This study demonstrates a statistically significant difference between afamin serum concentrations in pregnant women with pathologic OGTT results and those with normal 75-g OGTT in the second trimester. Patients with GDM requiring insulin showed slightly higher afamin serum concentrations as patients treated with diet. These findings were also observed in blood samples collected in the first trimester of women developing GDM during their ongoing pregnancy. Patients with subsequent iGDM in their later pregnancy exhibited higher afamin concentrations during the first trimester than women free of GDM or women with later GDM not requirering insulin therapy.

Afamin indicates IR, the pathophysiological key mechanism underlying GDM, which is linked to oxidative stress. The results of this pilot study indicate a promising role of a novel biomarker for pathological glucose metabolism in pregnancy.

Further research is recommended for determining afamin concentrations prospectively during the first trimester of pregnancy and correlating them with the results of 75-g OGTT during the second trimester. Additionally, studies involving larger populations are recommended for determining whether afamin concentrations are superior to the 50-g OGTT as a screening test that can determine which women are at high risk of GDM. An interesting question that remains unanswered is whether elevated mid-trimester serum concentrations of afamin are associated with adverse pregnancy outcomes. Future research is warranted in this area and may have an impact on how we manage pregnancies at risk to develop GDM.

## Abbreviations

CI: Confidence interval
DDG: Deutsche Diabetesgesellschaft
dGDM: Gestational diabetes mellitus treated with diet
DGGG: Deutsche Gesellschaft für Gynäkologie und Geburtshilfe
GDM: Gestational diabetes mellitus
HOMA: Homeostasis-Model Assessment
IADPSG: International Association of the Diabetes and Pregnancy Study Groups
iGDM: Insulin dependent gestational diabetes mellitus
IQR: Interquartile range
IR: Insulin resistance
OGTT: Oral glucose tolerance test
PCOS: Polycystic ovary syndrome
SHBG: Sexual hormone binding protein
TNF: Tumor-necrosis-factor
